# Effect of Filler Type, Content, and Silanization on the Flexural Strength, Elastic Modulus, Shore D Hardness, and Two-Body Wear of PAEK Compounds

**DOI:** 10.3390/ma18122736

**Published:** 2025-06-11

**Authors:** Felix Schmeiser, Wofgang Schramm, Felicitas Mayinger, Uwe Baumert, Bogna Stawarczyk

**Affiliations:** 1 Dental Materials Unit, Department of Prosthetic Dentistry, University Hospital, LMU Munich, 80336 Munich, Germany; felix.schmeiser@med.uni-muenchen.de (F.S.); felicitas.mayinger@med.uni-muenchen.de (F.M.); 2 Department of Chemical Development, bredent GmbH & Co. KG, 89250 Senden, Germany; wolfgang.schramm@bredent.com; 3 Mechanobiology and Genetics Research Area, Department of Orthodontics and Dentofacial Orthopedics, University Hospital, LMU Munich, 80336 Munich, Germany; uwe.baumert@med.uni-muenchen.de

**Keywords:** PAEK compounds, flexural strength, elastic modulus, shore D hardness, two-body wear, silica, filler

## Abstract

The aim of this study was to evaluate the influence of filler type, filler content, and filler silanization on the flexural strength (FX), elastic modulus (E_m_), shore D hardness (SDH), and two-body wear (2BW) of polyaryletherketone (PAEK) compounds. Specimens (40 wt% PEEK, 40 wt% PEK) with different filler types: 20 wt%: fumed silica (FS), calcium silicate (CS), feldspar (FP), magnesium silicate hydrate (MSH), no filler (NF); different filler content: 20, 25 or 30 wt% CS; different filler silanization: 20 wt% CS silanized with alkylsilane/aminosilane, FP silanized with methylsilane/ vinylsilane, no silanization; and PEEK_20_ (BioHPP) or PEEK_25_ (BioHPP plus) controls were fabricated and tested for FX, E_m_, and SDH. Two-body wear (4 × 100,000 cycles, 50 N, 2.5 Hz) with composite resin antagonists was measured with PAEK_i_ (35 wt% PEEK, 35 wt% PEK, 30 wt% CS), PAEK_ii_ (70 wt% PEEK, 30 wt% CS), PAEK_iii_ (70 wt% PEEK, 25 wt% CS, 5 wt% FP), and PEEK_20_ controls. Data were analyzed with Kolmogorov–Smirnov-, Kruskal–Wallis-H-, post hoc Scheffé test, pairwise comparisons, Bonferroni correction, one-way ANOVA, and Spearman rho (α = 0.05). An abrasion area analysis was performed. Adding filler increased FX, E_m_, and SDH, with CS and MSH showing the highest values for FX and E_m_. Adding 30 wt% CS increased FX, E_m_, and SDH compared with 20 wt%. Silanization with methylsilane increased FX, E_m_, and SDH. Silanization with aminosilane increased FX and SDH. PEEK_20_ showed the lowest 2BW compared with all EPCs. No material losses were detected on the antagonists. PAEK compounds with 25 to 30 wt% CS increased FX and E_m_ compared to lower contents, no filler, or PEEK_20_. Higher values of FX and E_m_ did not lead to lower 2BW.

## 1. Introduction

The fabrication of fixed and temporary dental prostheses (FDPs/TDPs) that integrate seamlessly into the stomatognathic system continues to pose a major challenge in dentistry [1,2]. Recently, interest in high-performance polymers (HPPs), particularly polyaryletherketones (PAEKs), which offer mechanical stability and esthetics while reducing rehabilitation costs, has been growing [2,3,4,5,6]. PAEKs include varying compositions of materials such as polyetheretherketone (PEEK), polyetherketoneketone (PEKK), and polyetherketone (PEK) [2,5,6]. PEEK offers a high flexural strength (FX; 140 to 170 MPa) [7], chemical resistance, opacity, and biocompatibility [8]. Its elastic modulus (E_m_) is similar to that of human bone (3 to 4 GPa), making it a promising alternative as a framework for interim and definitive FDPs and TDPs [8,9,10,11,12,13]. PEEK allows favorable stress absorption in abutment teeth, adjacent soft tissues, and the cementation layer [7,14,15]. PEKK has been reported to provide better shock absorbance than PEEK, with similar chemical, optical, and mechanical properties [16]. PEK has improved tensile strength and impact resistance with E_m_ comparable with that of PEEK [2,3,4,5]. The use of PAEKs as FDPs has been described, with a damping effect associated with the low E_m_ [7,17,18].

By incorporating fillers such as silica (SiO_2_), quartz, ceramic, and natural minerals, PAEKs can be optimized [12,19,20,21] with enhanced FX, E_m_, hardness, biocompatibility, and esthetics according to the type and content of the filler material [1,18,22,23,24,25]. Nano SiO_2_ has been successfully incorporated at 10 wt% into PEEK compounds, leading to improved FX, E_m_, and SDH [26,27,28], but this is reversed when more than 10 wt% is added [26]. Others have reported increased FX with 40 wt% SiO_2_ but decreased mechanical properties with less than 60 wt% of microbarium–aluminum borosilicate [23]. Martens hardness has been reported to be improved in PEEK compounds with increasing percentages of TiO_2_ [24]. Implant-supported four-unit PEEK FDPs have been reported to have better mechanical stability with a higher percentage of inorganic fillers [29]. However, even with filler, satisfactory esthetic and clinical outcomes cannot be achieved in monolithic restorations, as they exhibit a high opacity, grayish whitish color, and low translucency compared with those of dental ceramics and low mechanical properties such as an E_m_ lower than dentin (16 to 20 GPa) or enamel (48 to 105 GPa) [7,16,17,30,31,32,33].

Another key factor in optimizing the properties of PAEKs is the enhanced bond strength of filler particles inside the matrix through surface pretreatment such as silanization [21,26,34,35,36,37]. FX was enhanced with bifunctional SCA molecules such as (3-methacryloxypropyl)trimethoxysilane and (3-aminopropyl)triethoxysilane [38,39]. In dental adhesives, the durability at the composite–tooth interface could be increased by adding aminosilane [40,41,42,43]. However, research on the effect on the bonding strength to the polymer matrix is limited [26].

Two-body wear is a critical factor for dental restorative materials, as a rapid loss of the occlusal vertical dimension can affect the stomatognathic system, resulting in dentoalveolar compensation or the supra-eruption of teeth [26,44,45]. Adding nanohydroxyapatite fibers in an acrylate compound of bisphenol A diglycidyl methacrylate (bisGMA) and triethylene glycol dimethacrylate (TEGDMA) [46] or an acrylate blend of bisGMA and TEGDMA with increasing filler content of hydroxyapatite or a combination of hydroxyapatite and silica (1:1) to 40 wt% could enhance abrasion resistance [26]. The addition of fillers with a smaller particle size has been reported to lead to higher wear resistance in HPP compounds than fillers with a larger particle size [24,47]. However, because filler type and composition vary, data on the mechanical properties and two-body wear are limited, and its use for monolithic FDPs and TDPs remains unclear.

To better assess the performance of PAEK compounds, this investigation aimed to examine the effect of filler type, content, and silanization on the FX, E_m_, and SDH of PAEK compound and to examine the two-body wear of experimental PAEK compounds (EPCs) compared with PEEK_20_ (BioHPP) and PEEK_25_ (BioHPP plus) as control groups. The null hypotheses were that FX, E_m_, and SDH would not be affected by the addition of fillers (1) or the filler content (2), that silanization of the filler particles would have no effect on FX, E_m_, or SDH (3), and that the composition of the PAEK compounds would have no effect on the two-body wear (4).

## 2. Materials and Methods

**Figure 1:**
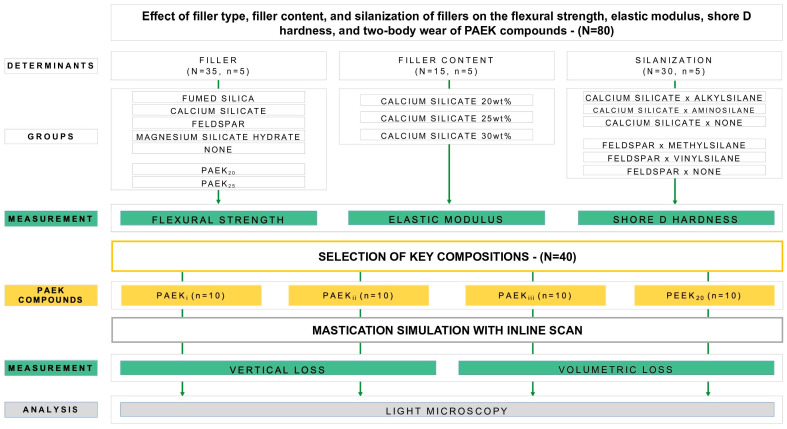
Study design—PAEK, polyaryletherketone; PEEK, polyetheretherketone.

PAEK flakes (50 wt% PEEK and 50 wt% PEK) were prepared by injection molding (Thermopress 400; bredent medical GmbH & Co. KG, Senden, Germany) at 400 °C, 20 MPa, with an injection speed of 17.5 mm/s (level 8) equivalent to an injection volume of 6.35 cm^3^/s, and a mold temperature of 200 °C. After predrying at 150 °C and grinding through a 3.0 mm sieve (SR 300; RETSCH, Haan, Germany), the PAEK flakes were processed to a dry blend with a particle size of d50 < 50 µm. According to the groups in Figure 1, filler was added to the dry blend and mixed in a drum roll mill (JEL FRM; J. Engelsmann AG, Ludwigshafen am Rhein, Germany).

**Figure 2:**
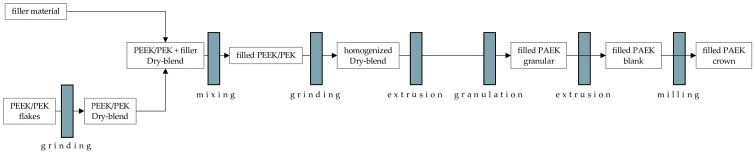
Fabrication process of PAEK specimens—PAEK, polyaryletherketone; PEEK, polyetheretherketone.

The PAEK dry blend was melted, dispersed, and extruded in a 16 mm twin-screw compounder (Eurolab 16; ThermoElectron, Langenselbold, Germany) into single-tooth blanks at a temperature of 380 °C and a speed of 450 rpm (Figure 2).

To investigate the influence of the filler, 20 wt% filler with an average particle size of d50 = 4 µm—namely fumed silica (FS), calcium silicate (CS), feldspar (FP), magnesium silicate hydrate (MSH), or no filler (NF)—was added (N = 35; n = 5) to the PAEK dry blend (Figure 1). For the effect of filler content, CS was added with 20 wt%, 25 wt%, or 30 wt% (N = 15, n = 5). For the effect of silanization, CS filler was silanized with alkylsilane, aminosilane, or not silanized (N = 15, n = 5), and FP filler was silanized with methylsilane, vinylsilane, or not silanized (N = 15, n = 5). EPC specimens (2 mm × 2 mm × 25 mm) were prepared according to the International Organization for Standardization ISO 10477 standard [48] from PAEK blends with 30 wt% of fillers, namely PAEK_i_, PAEK_ii_, and PAEK_iii_ (Table 1), and established PEEK compounds with 20 wt% (PEEK_20_; BioHPP; bredent medical GmbH & Co. KG) and 25 wt% (PEEK_25_; BioHPP Plus; bredent medical GmbH & Co. KG) of a ceramic filler.

FX (N = 45; n = 5) were tested with a universal testing machine (ZwickiLine Z2.5; ZwickRoell, Ulm, Germany). FX was calculated with the following formula:

(1) $$FX=\frac{3Fl}{2bh^{2}}$$

where *F* was the highest applied force, *l* the distance between the support bearings (20 mm), *b* the width, and *h* the height of the specimen. The traverse speed was 1 mm/min, support distance 20 mm, and radius of the support rolls 1 mm. E_m_ was detected at a strain between 0.1% and 0.15% with a displacement transducer. E_m_ was using the following:

(2) $$E_{m}=\frac{FX_{2}-FX_{1}}{0.0015-0.001}$$

SDH was measured in 4 mm × 50 mm × 40 mm specimens with a hand-held hardness tester (ZwickRoell 3130/31; ZwickRoell). The SDH was read after 3 s.

**Figure 3:**
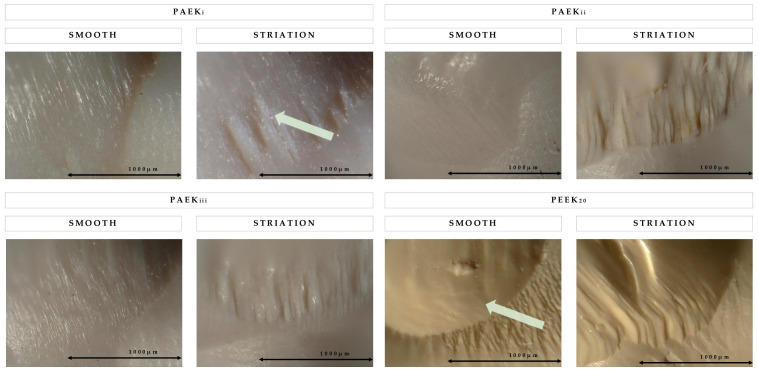
Microscope images of PAEK_i_, PAEK_ii_, PAEK_iii_, and PEEK_20_; (**left**): specimen with clear abrasion edge, (**right**): specimen with striations on abrasion edge; arrow PAEK_i_: chipped particles, arrow PEEK_20_: homogenous surface. Original magnification × 200.

Molar-shaped complete anatomic crowns [45] (N = 40, n = 10) were fabricated from 4 different EPCs, PAEK_i_, PAEK_ii_, PAEK_iii_, and PEEK_20_ as controls. The average particle size of the filler and the composition of the EPCs is shown in Table 1. Twenty-four hours before each mastication simulation test, both the surface of the abutments and the inside of the crowns were airborne abraded with 110 µm alumina powder (Korox 110; BEGO Bremer Goldschlägerei, Bremen, Germany) at a pressure of 0.15 MPa. The crowns were fixed on cobalt chromium abutments (CoCr; Ceramill Sintron; Amann Girrbach AG, Pforzheim, Germany) with a composite resin-based luting material (Solocem; Coltène/Whaledent AG, Altstätten, Switzerland) [45]. Antagonists made from composite resin (BRILLIANT Crios; Coltène/Whaledent AG) were milled to a hemisphere of Ø4 mm and embedded in a metal specimen holder with a casting resin (SCANDIQUICK; SCAN-DIA, Hagen, Germany) [45]. Mastication simulation was performed with a novel simulation and scanning device developed in a ZIM-cooperation (ZF4052008BA8) with equipment from SD-Mechatronik GmbH (Feldkirchen-Westerham, Germany) and bredent GmbH. Longitudinal data were generated with 400,000 cycles, a frequency of 2.5 Hz, a vertical load of 50 N, a vertical movement of 2 mm, and a lateral movement of 0.7 mm inline after every 100,000 cycles. The substrates were constantly wetted with distilled water at a temperature of 23 °C and automatically cleaned with oil-free air before each scan [45]. The abrasion surfaces were analyzed under an optical microscope (KEYENCE VHX-6000; KEYENCE Corp, Ôsaka, Japan) at ×200 magnification (Figure 3).

Data were analyzed with a statistical software program (IBM SPSS Statistics, v29.0; IBM Corp, Armonk, NY, USA). Descriptive statistics were reported with median, minimum and maximum, mean, standard deviation (SD), and 95% confidence intervals (95% CI) (α = 0.05). Deviation from the normality assumption was analyzed with the Kolmogorov–Smirnov test with normal distribution violated in fewer than 5% (filler, content, silanziation) and more than 5% (EPCs, two-body wear) of the test groups. Nonparametric results were analyzed with the Kruskal–Wallis H test, Scheffé post hoc tests, pairwise comparisons, and a Bonferroni correction. Parametric results were analyzed with one-way ANOVA and Bonferroni-corrected Scheffé tests. The correlation of vertical and volumetric loss was analyzed with the Pearson correlation (ρ).

## 3. Results

### 3.1. Effect of Filler Type on FX, E_m_, and SDH

**Figure 4:**
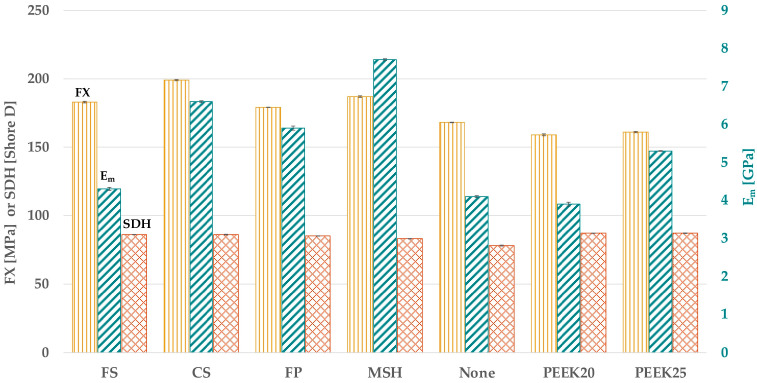
FX, E_m_, and SDH in dependence on the type of filler.

For the filler type, differences were observed among all test groups for each tested parameter (*p* < 0.001; Figure 4 and Table A1).

Comparing FX, PEEK_20_ and PEEK_25_ (*p* > 0.999) showed the lowest values compared to all other groups (*p* < 0.033). Adding no filler revealed a higher FX than PEEK_20_ and PEEK_25_ (*p* < 0.033) but lower values than adding FS (*p* < 0.033). The highest FX was detected for CS (*p* < 0.033) followed by MSH and FS (*p* < 0.033) with similar values (*p* > 0.999). FX with FP was lower than MSH (*p* < 0.033) but similar to FS (*p* > 0.999). PEEK_20_ showed the lowest E_m_ (*p* < 0.033), which was statistically similar to adding no filler (*p* > 0.999). FS showed an E_m_ similar to adding no filler (*p* > 0.999) but higher than PEEK_20_ (*p* < 0.033). The highest E_m_ was detected with MSH (*p* < 0.033), followed by CS (*p* < 0.033), FP (*p* < 0.033), and PEEK_25_ (*p* < 0.033), which showed a higher E_m_ than FS (*p* < 0.033). Considering the SDH, the lowest values were detected without a filler. Adding MSH revealed a higher SDH than no filler (*p* < 0.033) similar to CS (*p* = 0.206). The highest SDH was detected for PEEK_25_ (*p* < 0.033), followed by PEEK_20_, FS, CS, and FP with similar values (*p* > 0.206) but also with higher values than MSH (*p* < 0.033).

### 3.2. Effect of Filler Content on FX, E_m_, and SDH

**Figure 5:**
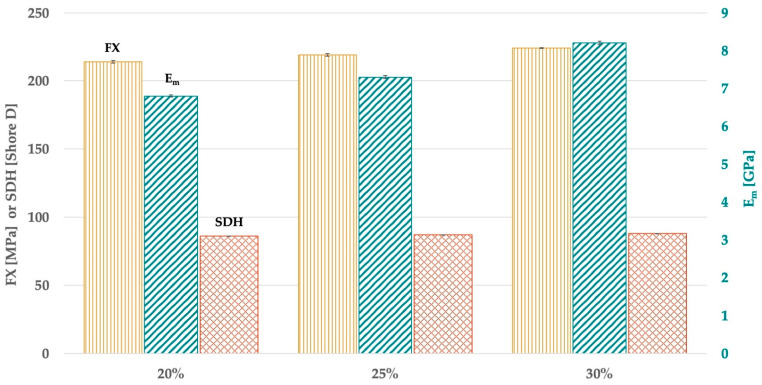
FX, E_m_, and SDH in dependence on the content of CS.

Comparing different filler contents of CS (Figure 5 and Table A1), a higher FX was found with a content of 30 wt% and 25 wt% (*p* = 0.152) compared with 20 wt% (*p < 0*.043). The highest values of E_m_ were measured with a content of 30 wt% (*p* < 0.001), followed by 25 wt% (*p* < 0.001) and 20 wt% (*p* < 0.001). Adding 25 wt% of CS revealed similar values of SDH as adding 20 wt% and 30 wt% (*p* > 0.061). Adding 30 wt% of CS showed a higher SDH than adding 20 wt% (*p* < 0.004).

### 3.3. Effect of Filler Silanization on FX, E_m_, and SDH

**Figure 6:**
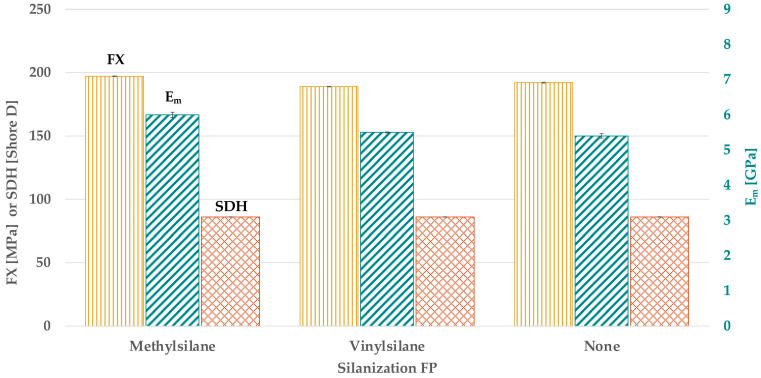
FX, E_m_, and SDH in dependence on the silanization of FP.

Considering the silanization of FP (Figure 6 and Table A2), methylsilane showed a higher FX and E_m_ than vinylsilane (*p* < 0.024) or no silanization (*p* < 0.043), with statistically similar values (*p > 0*.132).

**Figure 7:**
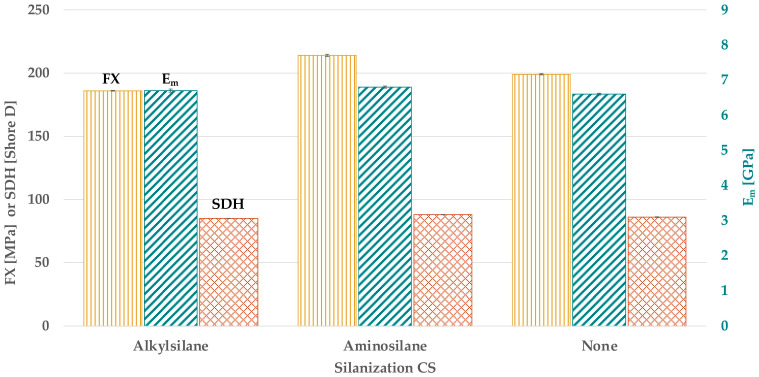
FX, E_m_, and SDH in dependence on the silanization of CS.

No statistical difference was found regarding SDH (*p* > 0.186). The silanization of CS (Figure 7 and Table A2) showed no statistical difference for E_m_ (*p* > 0.311). By using aminosilane, a higher FX than no silanization (*p* < 0.001) was found. By using alkylsilane, a lower FX than no silanization (*p* < 0.001) but a similar SDH (*p* = 0.362) was found. By using aminosilane, the highest SDH (*p < 0*.026) was found.

### 3.4. Mechanical Properties of Experimental PAEK Compounds and Effect of Two-Body Wear

**Figure 8:**
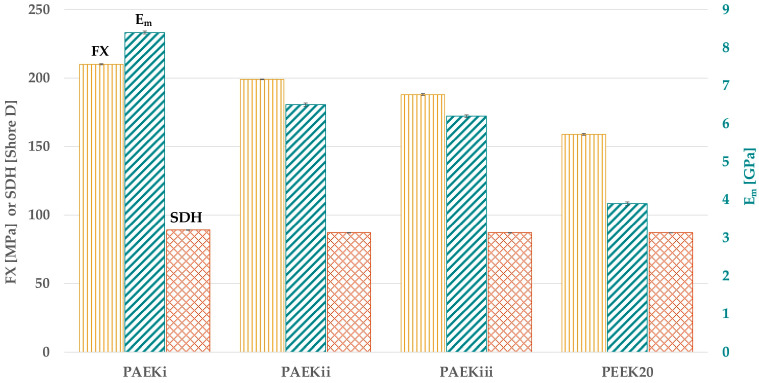
FX, E_m_, and SDH of EPCs and PAEK_20_.

Considering the mechanical properties of the EPCs (Figure 8 and Table A2), the highest FX was for PAEK_i,_ followed by PAEK_ii_ (*p* < 0.001), PAEK_iii_ (*p* < 0.001), and PEEK_20_ (*p* < 0.001). PAEK_i_ had the highest E_m_ (*p* < 0.001), followed by PAEK_ii_ and PAEK_iii_ with similar values (*p* = 0.098). PEEK_20_ had the lowest E_m_ (*p* < 0.001) compared with all EPCs. No significant differences were found regarding SDH (*p* > 0.999) between the EPCs and PEEK_20_.

**Figure 9:**
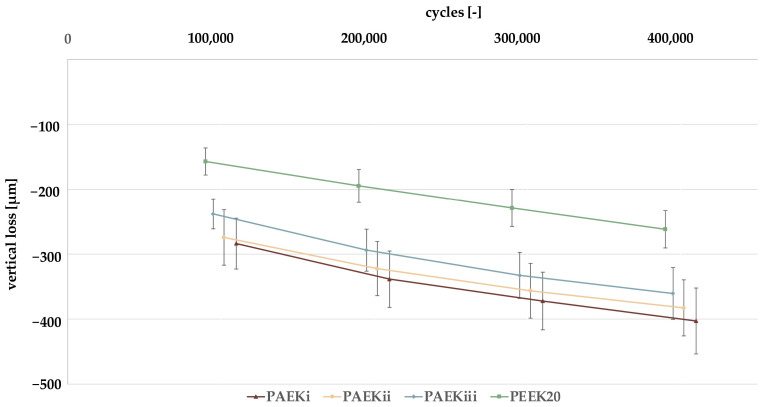
Vertical material losses [µm] within cycle intervals of 100,000 cycles—PAEK, polyaryletherketone; PEEK, polyetheretherketone.

A positive correlation between vertical and volumetric loss was observed for all cycle steps (*r*_100,000_ = 0.868, *p* < 0.001; *r*_200,000_ = 0.806, *p* < 0.001; *r*_300,000_ = 0.782, *p* < 0.001; *r*_400,000_ = 0.767, *p* < 0.001). Therefore, two-body wear results were described by using the vertical material losses (Figure 9, Table A3).

The number of cycles showed the highest effect size on the material losses (partial eta-squared (*η_p_*^2^) = 0.970, *p* < 0.001), followed by the material (*η_p_*^2^ = 0.947, *p* < 0.001). The interaction of cycle and material had no impact on the material losses (*p* = 0.260). Overall, an increasing value of material losses with an increasing number of cycles (*p* < 0.05) was detected. Regardless of the number of cycles, PEEK_20_ showed the lowest material losses (*p* < 0.001). Concerning the individual intervals of cycles, no differences were detectable among the EPCs *p* > 0.999) (Figure 9 and Table A3).

### 3.5. Qualitative Analysis of Abrasion Area

Considering the qualitative microscopy of the representative specimens (Figure 3), PAEK_i_ and PAEK_iii_ showed a fine structure with evenly parallel striations and a clear margin at the abrasion edge in 6 of 10 specimens. In 4 of 10 specimens, this clear margin was not homogeneous but had elevations. In PAEK_i_, these elevations were connected to the abrasion edge. PAEK_ii_ showed a homogeneous abrasion surface in 8 of 10 specimens, which only occasionally showed a fine structure. In 2 of 10 specimens, there were larger elevations with a clear abrasion edge. The abrasion surface of 8 of 10 specimens of PEEK_20_ was predominantly smooth and homogeneous, showing a structured surface outside the abrasion area. The edges were defined by a clear line. However, 2 of 10 specimens showed clear grooves on the edge and on the surface of the abrasion area.

## 4. Discussion

The aim of this investigation was to evaluate the effect of filler type, filler content, and silanization of fillers on FX, E_m_, SDH, and the two-body wear of PAEK compounds. The addition of filler and its content showed significant differences on FX, E_m_, and SDH; therefore, the null hypotheses that FX, E_m_, and SDH would not be affected by the addition of fillers (1) or the filler content (2) were rejected. The silanization of the filler particles led to an increased FX and E_m_; therefore, the null hypothesis that the silanization of the filler particles would have no effect on FX, E_m_, or SDH (3) was rejected. The composition of the EPCs had no effect on the two-body wear, since there was no significant difference in material loss; therefore, the null hypothesis that the composition of the PAEK compounds in terms of their matrix and the addition of fillers would have no effect on the two-body wear (4) was not rejected.

The influence of the filler type was investigated with a concentration of 20 wt% and a mean grain size of d50 = 4 µm [47]. FX showed the highest values for CS, followed by MSH and FS, consistent with the results of previous investigations [24,25,26]. Contrasting one of these [26], a weight fraction above 10 wt% did not lead to a reversal of FX, E_m_ or SDH. Despite the lower FX, MSH showed higher E_m_ than CS, FP, and PEEK_25_. Accordingly, different properties are affected depending on the filler type. Therefore, CS appears to particularly enhance FX and MSH appears to enhance E_m_. The filler content showed less of an effect on SDH, as PEEK_20_ and PEEK_25_ were both in the upper value range. Nevertheless, the addition of a filler increased the SDH significantly, regardless of the polymer matrix. Accordingly, CS, MSH, and FP fillers have the potential to provide improved stability, a property acknowledged in other studies as crucial yet currently insufficient for the successful implementation of monolithic FDPs [7,26,32]. However, these findings need further investigation. Concerning the filler content, lower values of FX were only observed at 20 wt% of CS, but E_m_ was constantly higher with increasing filler content [26,27,28,38]. Accordingly, the filler content could have a larger effect on E_m_. An increased density has been strongly connected to hardness [47]. The resistance to penetration increases with increasing filler content as the density of filler rises, since SDH was enhanced with 30 wt%. However, an increase in filler content and thus SDH would possibly be at the expense of FX and E_m_ [26,27,28,38]. Therefore, a higher filler content appears to increase the hardness of the PAEK compounds at 30 wt% [23]. Further investigations with filler contents are necessary [22,23,26]. Silanization is used as a bonding agent to increase the bonding strength between silica-based (or silica-coated) indirect restorations and resin composite [40]. Accordingly, the silanization of the filler can strengthen the bond to the polymer matrix and thus increase the FX and E_m_ of the PAEK compounds as seen in FP with methylsilane and CS with aminosilane [35,40]. The stronger bond is based on two kinds of functional groups, silane-reactive or hydrolyzable groups, showing affinity for the filler, and an organic chain ending in a functional group with chemical affinity to the polymer [41,42,43]. It appears to increase the resistance to stress and deformation. If CS is used, silanization with aminosilane could lead to a higher SDH against indentation. However, further investigations are required to clarify the effect of the filler-to-matrix connection and whether the types of fillers used, the range of filler contents, and the silanization agents are representative [26].

The in vitro mastication simulation was performed under clinically relevant conditions with anatomic crowns bonded on Co-Cr abutments with a self-adhesive and dual-polymerizing composite resin-based luting material. The temperature was held constantly at 23 °C as there was no difference compared to thermal cycles of 5 °C and 55 °C shown in previous investigations [45]. Although the E_m_ of Co-Cr abutments (200 GPa) is higher than of dentin (16 to 20 GPa) [30], abrasion values similar to those of dentin have been reported with polymer-based crowns [45]. The cusp inclination corresponded to the natural tooth to generate higher material losses to differentiate among the materials [45]. Enamel as an antagonist could lead to errors in standardization associated with variations in the donor, so composite resin was used, as it has been reported to behave similarly to natural teeth [45]. The authors are unaware of previous studies that have generated longitudinal data with inline scans of the material losses at an interval of 100,000 cycles each. Wear simulation and scanning performance were similar to an established mastication simulator (CS-4; SD-Mechatronik) and a laser scanner (LAS 20; SD-Mechatronik) as investigated in a ZIM-cooperation project (ZF4052008BA8). A matrix of PEEK was used for both the EPCs and PEEK_20,_ with PAEK_i_ containing equal parts of PEEK and PEK. Based on the previous results, CS with d50 = 4 µm [24] provided increased FX and E_m_ compared with the other fillers tested in the present study. FP with d50 = 1 µm was added to PAEK_iii_, as it is nontoxic and increases the resistance to compressive forces and wear associated with the smaller grain size [24,25,47]. Although the EPCs varied in their compositions of filler and matrix, no difference in any cycle interval was detected. Thus, it was concluded that the composition in the tested range of compositions had no effect on two-body wear, reflected in similar abrasion areas (Figure 3). When the antagonist is impacted, good resistance is expected, with horizontal movement causing distant material to be pushed along the abrasion surface. The abrasion of the material causes the particles to agglomerate, be torn from the microstructure, and be transported with the antagonist. In the process, some of the particles become attached between the crown and the antagonist and, because of their higher E_m_, lead to areas with increased material loss.

Comparing the mean values of the material loss from the PAEK composites, two-body wear tended to decrease from PAEK_i_ to PAEK_ii_ and PAEK_iii_, whereby the difference after 100,000 cycles from PAEK_i_ to PAEK_ii_ (∆ = 10 µm) was smaller compared with PAEK_ii_ to PAEK_iii_ (∆ = 36 µm). Therefore, the type of particle size distribution and the addition of PEK to PAEK_i_ appears to have a minor influence on two-body wear. Also, the addition of FP with a smaller particle size (d50 = 1 µm) to PAEK_iii_ appeared to lower two-body wear, as reported previously [24,25,47]. As shown in recent investigations, improved wear is a key factor for PAEK to use as monolithic FDP [7,15]. A smaller particle size in the range of d50 = 1 µm could increase the abrasion resistance of FDPs and TDPs as they provide a more homogenous distribution of stress and tend to have a stronger and more homogenous bonding to the matrix [7]. As seen in Figure 3, PAEK_i_ tends to release larger particles on the striation areas, increasing stress during abrasion, which leads to a larger chipping of material compared to PAEK_ii_ and PAEK_iii_. However, further investigations are required with varying filler types and particle sizes. Compared with the material loss of established materials, the values of the EPCs after 100,000 cycles are similar to those of compomer (306 µm) (Compoglass F; Ivoclar AG, Schaan, Liechtenstein), microhybrid composite resin (181 µm) (Arabesk; VOCO, Cuxhaven, Germany) and nanohybrid composite resin (205 µm) (Tetric Ceram; Ivoclar AG) [34]. Therefore, the EPCs may be suitable for an FDP based on the results of two-body wear. However, these studies were carried out after 120,000 cycles with a Ø6 mm steatite antagonist, which limits comparability [24]. In addition, its suitability as an FDP or TDP material must be confirmed by further investigations with a higher number of cycles and varying compositions. The size of the ceramic particles in PEEK_20_ is approximately 0.3 to 0.5 µm, resulting in a fine polymer structure optimizing its mechanical properties, with E_m_ significantly lower than for the other EPCs [25]. This is consistent with the present results, as material losses were lower for PEEK_20_ compared with the EPCs at all intervals. Accordingly, it was concluded that smaller particle sizes led to a higher abrasion resistance consistent with previous studies [25,34,47]. However, the 30 wt% EPCs showed higher FX and E_m_. As the mastication simulation is a dynamic load with a vertical track, the high filler contents could lead to an increased formation of agglomerates, particularly with smaller particle sizes, which reduces the homogeneity and structure of the material. When observing the abrasion surface with a light microscope, PEEK_20_ had a smooth surface at the impact point of the antagonist. The lower E_m_ in PEEK_20_ may have led to the deformation of the material with a damping effect [18], which could partly compensate for the abrasive precedents leading to reduced material losses. Additionally, the abrasion surfaces of PEEK_20_ are more homogenous than those of the EPCs, resulting in less instances of structures chipping. Concerning the application of PAEK compounds as monolithic FDPs or TDPs, the use of finer particles could lead to lower two-body wear, which, however, would have to be examined by further investigations with various combinations of fillers, contents, and silanization. Further, the results of the present investigation must yet be confirmed by long-term and clinical studies.

The present investigation is limited by the used fillers, contents, and silanization substances as well as the manufacturing process of the PAEK flakes and fillers, which could have affected the dispersion compared to the established materials PEEK_20_ and PEEK_25_. Due to the different density of the fillers, the volumetric fraction of the EPCs could be different for the same mass fraction. Future studies could benefit from a volume-based formulation to gain further knowledge of the influence of fillers.

One further limitation is that no power analysis was performed a priori to determine an adequate sample size. For all tested parameters, a post hoc power analysis (R-Version 4.2.1 (RStudio 2025.05.0 Build 496), RStudio, Boston, MA, USA) was performed. For a sample size of n = 5, the lowest two-sided, two-sample t-test power was equal to 99.86% for E_m_ of aminosilane and alkylsilane, with an observed effect of 0.2 GPa and a pooled SD of 0.055. For a sample size of n = 10, the lowest two-sided, two-sample t-test power was equal to 100% for E_m_ of PAEK_i_ and PEEK_20_, with an observed effect of 0.2 GPa and a pooled SD of 0.055. In addition, it is limited by the composition of the PAEK compounds, number of cycles, and the design of the crown. Further studies are necessary to verify the observed results and address other determinants such as the filler dispersion as well as detailed chemical analyses with other compositions of PAEK compounds, dental materials, and numbers of cycles.

## 5. Conclusions

Based on the findings of this in vitro study, the following conclusions were drawn:

1. Silica-based fillers at 20 wt% led to increased flexural strength, elastic modulus, and shore D hardness compared with no filler, which could provide sufficient mechanical properties for monolithic fixed prostheses.
2. The filler content had a stronger effect on the elastic modulus than on flexural strength.
3. Silanization of calcium silicate with aminosilane led to increased mechanical properties.
4. Smaller particle sizes and lower elastic modulus led to a higher abrasion resistance.

## Figures and Tables

**Table 1:** Compositions of experimental PAEK compounds PAEK_i_, PAEK_ii_, and PAEK_iii_ (wt%).

Components	PAEK_i_	PAEK_ii_	PAEK_iii_
PEEK	35	70	70
PEK	35	0	0
Calcium silicate (d50 = 4 µm); wide particle size distribution	30	0	0
Calcium silicate (d50 = 4 µm); narrow particle size distribution	0	30	25
Feldspar d50 = 1 µm	0	0	5

## Data Availability

The original contributions presented in this study are included in the article. Further inquiries can be directed to the corresponding author.

## Abbreviations

The following abbreviations are used in this manuscript:

2BW	Two-body wear
CS	Calcium silicate
E_m_	Elastic modulus
EPC	Experimental PAEK compounds
FDP	Fixed dental prostheses
FP	Feldspar
FS	Fumed silica
FX	Flexural strength
MSH	Magnesium silicate hydrate
NF	No filler
PAEK	Polyaryletherketones
PEEK	Polyetheretherketone
PEEK_20_	BioHPP
PEEK_25_	BioHPP plus
PEK	Polyetherketone
PEKK	Polyetherketoneketone
SDH	Shore D hardness
TDP	Temporary dental prostheses

## Appendix A

**Table A1:** Descriptive statistics of FX, E_m_, and SDH in dependance on the type of filler and filler content (CS); minimum, median, maximum, mean ± standard deviation (SD), and 95% confidence intervals (CI).

Groups	Flexural Strength [MPa]		E-Modulus [GPa]		Shore Hardness [Shore D]	
	Min/Median/Max **	Mean ± SD (95% CI)	Min/Median/Max **	Mean ± SD (95% CI)	Min/Median/Max **	Mean ± SD (95% CI)
Type of filler *						
FS	179/184/186 ^c,d^	183 ± 1.2	4.2/4.2/4.6 ^b^	4.3 ± 0.08	85/86/86 ^c^	86 ± 0.2
		(179;187)		(4.1;4.5)		(84;87)
CS	196/199/201 ^e^	199 ± 0.9	6.5/6.6/6.8 ^e^	6.6 ± 0.05	84/86/87 ^b^	86 ± 0.5
		(195;202)		(6.5;6.8)		(83;88)
FP	178/179/180 ^c^	179 ± 0.3	5.5/5.9/6.1 ^d^	5.9 ± 0.11	85/85/86 ^c^	85 ± 0.2
		(177;180)		(5.5;6.2)		(83;86)
MSH	183/187/190 ^d^	187 ± 1.2	7.5/7.7/7.8 ^f^	7.7 ± 0.05	82/83/84 ^b^	83 ± 0.3
		(182;191)		(7.5;7.8)		(81;84)
None	168/168/170 ^b^	168 ± 0.4	3.9/4.0/4.2 ^a,b^	4.1 ± 0.05	76/78/80 ^a^	78 ± 0.7
		(166;170)		(3.9;4.2)		(75;81)
PEEK_20_	154/160/162 ^a^	159 ± 1.4	3.6/3.9/4.1 ^a^	3.9 ± 0.10	86/87/87 ^c^	87 ± 0.2
		(154;163)		(3.6;4.1)		(84;88)
PEEK_25_	157/161/163 ^a^	161 ± 1.0	5.3/5.3/5.4 ^c^	5.3 ± 0.02	86/87/88 ^d^	87 ± 0.4
		(156;164)		(5.3;5.4)		(85;89)
Filler content *						
20%	207/215/217 ^a^	214 ± 1.7	6.7/6.7/6.9 ^a^	6.8 ± 0.06	84/86/87 ^a^	86 ± 0.5
		(207;219)		(6.6;6.9)		(83;88)
25%	214/219/223 ^b^	219 ± 1.7	7.1/7.3/7.5 ^b^	7.3 ± 0.07	86/87/88 ^a,b^	87 ± 0.4
		(213;225)		(7.1;7.5)		(85;89)
30%	222/224/225 ^b^	224 ± 0.5	8.0/8.1/8.5 ^c^	8.2 ± 0.09	87/88/89 ^b^	88 ± 0.3
		(221;226)		(8.0;8.5)		(86;89)

* = normal distribution; ** small letters: groups within FX, E_m_ or SDH.

**Table A2:** Descriptive statistics of FX, E_m_, and SDH in dependance on the silanization (CS) and mechanical properties; minimum, median, maximum, mean ± SD, and 95% (CI).

Silanization FP *	Flexural Strength [MPa]		E-Modulus [GPa]		Shore Hardness [Shore D]	
	Min/Median/Max **	Mean ± SD (95% CI)	Min/Median/Max **	Mean ± SD (95% CI)	Min/Median/Max **	Mean ± SD (95% CI)
Methylsilane	195/197/198 ^b^	197 ± 0.5 (194;199)	5.6/6.0/6.5 ^b^	6.0 ± 0.15 (5.6;6.4)	85/86/86 ^a^	86 ± 0.2 (83;87)
Vinylsilane	188/189/190 ^a^	189 ± 0.4 (187;191)	5.4/5.5/5.5 ^a^	5.5 ± 0.03 (5.4;5.5)	85/86/87 ^a^	86 ± 0.3 (84;87)
None	190/192/194 ^a^	192 ± 0.7 (188;194)	4.9/5.4/5.6 ^a^	5.4 ± 0.13 (5.0;5.7)	85/86/87 ^a^	86 ± 0.4 (84;88)
Silanization CS *						
Alkylsilane	185/186/188 ^a^	186 ± 0.5 (183;188)	6.6/6.7/7.1 ^a^	6.7 ± 0.09 (6.5;7.0)	84/85/85 ^a^	85 ± 0.2 (83;86)
Aminosilane	207/215/217 ^c^	214 ± 1.7 (207;219)	6.7/6.7/6.9 ^a^	6.8 ± 0.06 (6.6;6.9)	87/88/88 ^b^	88 ± 0.2 (86;89)
None	196/199/201 ^b^	199 ± 0.9 (195;202)	6.5/6.6/6.8 ^a^	6.6 ± 0.05 (6.5;6.8)	84/86/87 ^a^	86 ± 0.5 (83;88)
Experimental PAEK compounds (EPCs)						
PAEK_i_	208/210/213 ^d^	210 ± 0.8 (207;213)	8.1/8.4/8.6 ^c^	8.4 ± 0.08 (8.2;8.6)	88/89/90 ^a^	89 ± 0.4 (87;90)
PAEK_ii_	198/199/200 ^c^	199 ± 0.4 (197;201)	6.3/6.6/6.8 ^b^	6.5 ± 0.09 (6.2;6.8)	86/87/88 ^a^	87 ± 0.4 (86;88)
PAEK_iii_	185/189/193 ^b^	188 ± 1.5 (183;193)	6.0/6.2/6.4 ^b^	6.2 ± 0.07 (6.0;6.4)	86/87/88 ^a^	87 ± 0.4 (85;87)
PEEK_20_	154/160/162 ^a^	159 ± 1.4 (155;162)	3.6/3.9/4.1 ^a^	3.9 ± 0.09 (3.6;4.1)	86/87/87 ^a^	87 ± 0.2 (85;87)

* = normal distribution; ** lowercase letters: groups within FX, E_m_ or SDH. PAEK, polyaryletherketone; PEEK, polyetheretherketone.

**Table A3:** Descriptive statistics of vertical loss after two-body wear simulation with minimum, median, maximum, mean ± standard deviation (SD), and 95% confidence intervals (CI).

	Vertical Loss [µm]							
	100,000 Cycles		200,000 Cycles		300,000 Cycles		400,000 Cycles	
	Min/Median/Max *	Mean ± SD (95% CI)	Min/Median/Max *	Mean ± SD (95% CI)	Min/Median/Max *	Mean ± SD (95% CI)	Min/Median/Max *	Mean ± SD (95% CI)
PAEK_i_	−152/−302 /−371 ^b^	−284 ± 25 (−226;−340)	−189/−367 /−443 ^b^	−338 ± 27 (−275;−401)	−205/−408 /−471 ^b^	−372 ± 28 (−307;−436)	−212/−436 /−513 ^b^	−403 ± 32 (−330;−475)
PAEK_ii_	−178/−252 /−423 ^b^	−274 ± 27 (−212;−336)	−234/−301 /−481 ^b^	−322 ± 26 (−261;−382)	−267/−331 /−511 ^b^	−356 ± 27 (−294;−417)	−282/−363 /−535 ^b^	−383 ± 27 (−319;−445)
PAEK_iii_	−175/−236 /−297 ^b^	−238 ± 15 (−204;271)	−200/−285 /−375 ^b^	−294 ± 20 (−246;−341)	−235/−330 /−424 ^b^	−333 ± 22 (−280;−384)	−256/−359 /−469 ^b^	−361 ± 25 (−302;−418)
PEEK_20_	−105/−175 /−216 ^a^	−157 ± 13 (−126;−187)	−135/−201 /−269 ^a^	−195 ± 16 (−157;−231)	−167/−220 /−319 ^a^	−229 ± 18 (−187;−270)	−208/−235 /−360 ^a^	−262 ± 18 (−219;−303)

* lowercase letters: groups within one cycle interval. PAEK, polyaryletherketone; PEEK, polyetheretherketone.
